# Hyperthyroidism after radiofrequency ablation for thyroid nodule

**DOI:** 10.1002/kjm2.12809

**Published:** 2024-02-19

**Authors:** How‐Chen Wang, Che‐Wei Wu, Tzu‐Yen Huang

**Affiliations:** ^1^ School of Medicine, College of Medicine, Kaohsiung Medical University Kaohsiung Taiwan; ^2^ Department of Otorhinolaryngology–Head and Neck Surgery, International Thyroid Surgery Center Kaohsiung Medical University Hospital, Kaohsiung Medical University Kaohsiung Taiwan; ^3^ Department of Otorhinolaryngology, School of Post‐Baccalaureate Medicine and School of Medicine College of Medicine, Kaohsiung Medical University Kaohsiung Taiwan

Radiofrequency ablation (RFA) is a novel treatment for selected thyroid nodules and is known for its low complication rate when performed precisely.^1^, ^2^ Hyperthyroidism typically occurs within 2–4 weeks post‐RFA, is transient and self‐limiting and is hypothesized to be caused by destructive thyroiditis.^1^ We present a case of hyperthyroidism that developed 3 months post‐RFA.

A 41‐year‐old Taiwanese woman presented with a movable midline neck mass and mild compressive symptoms without tenderness or thyrotoxic signs. She smoked a pack daily and denied past irradiation, a history of thyroid disease, or any significant family history.

Thyroid sonography revealed a low‐risk 3.20 × 2.62 × 3.63 cm left thyroid nodule with peripheral vascularity (Figure 1A) and an unremarkable right lobe. Two fine‐needle aspirations showed a benign cytology. Her serum‐free T4, thyroglobulin (Tg), and Tg antibody levels were normal, in contrast with her low TSH and high microsomal Ab levels. After shared decision‐making and informed consent, the patient elected to undergo RFA.

**FIGURE 1:**
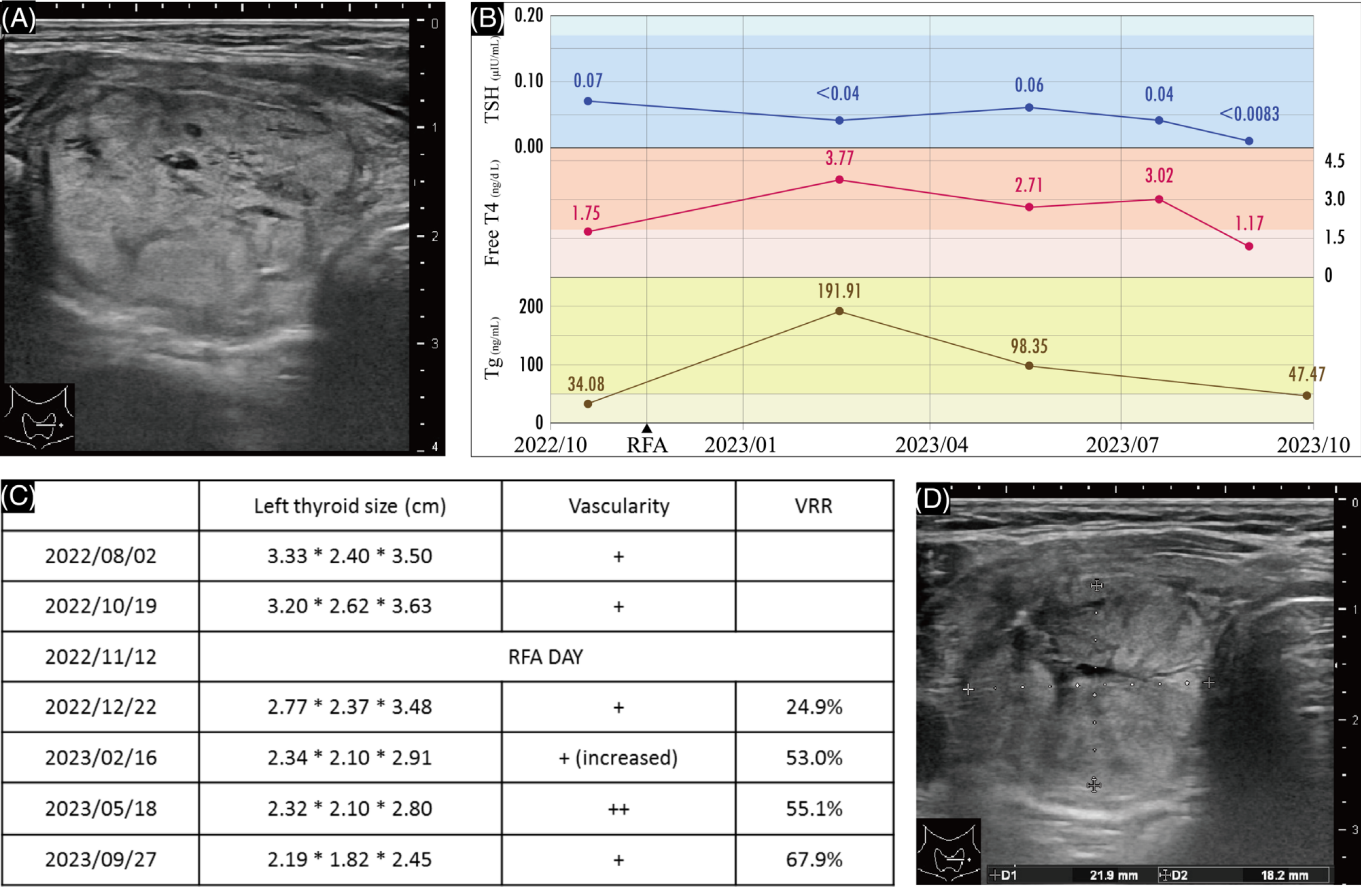
(A) Pre‐radiofrequency ablation (RFA) sonography revealed a 3.20 × 2.62 × 3.63 cm left thyroid nodule. (B) Free T4, TSH, and thyroglobulin (Tg) levels over time with reference values in lighter‐colored blocks. (C) Thyroid nodule size, vascularity, and volume reduction rate (VRR) were demonstrated. *Vascularity, “+” for peripheral, “++” for central vascularity under 50% of the volume. (D) Post‐RFA sonography at 46 weeks revealed the nodule measuring 2.19 × 1.82 × 2.45 cm.

Ultrasound‐guided RFA was performed on November 12, 2022, using 30–40 W power for 557 s of ablation activation time, delivering a total energy of 13,810 W s. The patient was discharged the next day with no discomfort.

Post‐RFA, routine laboratory tests at 14 weeks revealed elevated free T4 and Tg and decreased TSH (Figure 1B). The patient experienced palpitations and cold sweats for more than a month. Sonography at 6, 14, and 27 weeks showed nodule shrinkage but increased vascularity, particularly central vascularity, at 27 weeks (Figure 1C). As her hyperthyroidism progressed, 15 mg/day of methimazole was initiated at Week 28, and the dosage was increased to 20 mg/day during Week 33. By Week 42, her hyperthyroidism had completely resolved. The volume reduction rate at Week 46 was 67.9%. (Figure 1D).

In summary, the patient was initially euthyroid with positive microsomal antibodies. Three months post‐RFA, she developed hyperthyroidism and increased nodule vascularity as the nodule decreased in size.

RFA complications range from pain, skin burns, and hematoma formation to adjacent structural injuries and nodule rupture.^3^, ^4^ Thyroid dysfunction in the long term was documented, in which patients with diabetes or lower baseline TSH levels are prone to thyrotoxicosis.^4^

In the current literature, only one case of post‐RFA hyperthyroidism requiring treatment was documented by McAninch et al. In this case, symptomatic hyperthyroidism developed 8 weeks post‐RFA and was later diagnosed as Graves' disease requiring thyroidectomy.^5^ In comparison, our patient developed hyperthyroidism 14 weeks post‐RFA, which persisted for 10 months and was successfully treated with methimazole to avoid thyroidectomy.

Previous studies suggested that transient thyrotoxicosis post‐RFA may be caused by destructive thyroiditis.^1^ However, our patient's later onset and longer duration suggest a greater likelihood of autoimmunity. Further research is needed to clarify the pathophysiology involved.

Several points can be considered to improve future management. First, earlier thyroid function follow‐up than 14 weeks post‐RFA could better pinpoint hyperthyroidism onset and improve outcomes. Second, while methimazole successfully controlled the patient's hyperthyroidism, its effect on reducing the volume or regrowth risk has not been determined. Third, patients with transient thyroid dysfunction related to stressful events may have similar thyroid laboratory profiles as those in this case, and the corresponding clinical presentation can be further analyzed. Finally, managing autoimmune thyroiditis or Graves' disease pre‐RFA and post‐RFA and understanding changes in vascularity need to be considered.

In conclusion, hyperthyroidism is a potential complication in patients with underlying thyroiditis who are receiving RFA. Physicians must be aware of this risk, adequately inform patients, and carefully monitor thyroid function before and after the procedure.

## FUNDING INFORMATION

This study was supported by grants from Kaohsiung Medical University Hospital, Kaohsiung Medical University (KMUH111‐1R48; KMUH112‐2R51), and National Science and Technology Council (112‐2314‐B‐037‐034‐), Taiwan.

## CONFLICT OF INTEREST STATEMENT

All authors declare no conflict of interest.
